# Peer Recruitment Strategies for Female Sex Workers Not Engaged in HIV Prevention and Treatment Services in Côte d’Ivoire: Program Data Analysis

**DOI:** 10.2196/18000

**Published:** 2020-10-01

**Authors:** Oluwasolape Olawore, Hibist Astatke, Tiffany Lillie, Navindra Persaud, Carrie Lyons, Didier Kamali, Rose Wilcher, Stefan Baral

**Affiliations:** 1 Department of Epidemiology Johns Hopkins School of Public Health Baltimore, MD United States; 2 FHI 360/LINKAGES Washington, DC United States; 3 FHI 360/LINKAGES Abidjan Cote D'Ivoire; 4 FHI 360/LINKAGES Durham, NC United States

**Keywords:** female sex workers, HIV, Côte d’Ivoire, programmatic data, peer referral

## Abstract

**Background:**

In the context of the mostly generalized HIV epidemic in Côte d’Ivoire, key populations bear a higher burden of HIV than that borne by the general reproductive-aged population. Mathematical models have demonstrated the significant potential impact and cost-effectiveness of improving the coverage of HIV prevention and treatment services for key populations in Côte d’Ivoire. However, in 2019, coverage of these services remained limited by multiple intersecting stigmas affecting key populations, necessitating the study of innovative implementation strategies to better meet the needs of those most marginalized. Here, we leverage programmatic data to compare the effectiveness of the enhanced and traditional peer outreach approaches in reaching and providing community HIV testing to female sex workers not readily engaged in HIV prevention and treatment services in Côte d’Ivoire.

**Objective:**

The aim of this study was to describe the characteristics of female sex workers reached by the LINKAGES project in Côte d’Ivoire with enhanced peer outreach and traditional peer outreach and to compare HIV-related outcomes between the women reached by both strategies.

**Methods:**

Deidentified routine programmatic data collected as part of LINKAGES Côte d’Ivoire between October 2017 and April 2018 were used in these analyses. Demographic characteristics and HIV indicators including HIV testing history, HIV case-finding, linkage to HIV treatment, and treatment initiation were assessed using descriptive statistics. Differences in these indicators were compared by outreach strategy using Pearson chi-square tests.

**Results:**

There were 9761 women reached with enhanced peer outreach and routine peer outreach included in these analyses. The overall case-finding rate in the sample was 7.8% (698/8851). Compared with women reached by routine outreach, those reached by enhanced peer outreach were more likely to have previously been tested for HIV (enhanced: 1695/2509, 67.6%; routine: 4302/7252, 60.0%; χ^2^_1_=43.8; *P*=.001). The enhanced peer outreach approach was associated with a higher HIV case-finding rate (enhanced: 269/2507 10.7%; routine: 429/6344, 6.8%; χ^2^_1_=32.3; *P*=.001), higher proportion of linkage to treatment (enhanced: 258/269, 95.9%; routine: 306/429, 71.3%; χ^2^_1_=64.4; *P*=.001), and higher proportion of treatment initiation (enhanced: 212/269, 78.8%; routine: 315/429, 73.3%; χ^2^_1_=2.6; *P*=.11). Women reached by both approaches were categorized as high risk for HIV-related behaviors such as condomless sex and number of sex acts in the previous week.

**Conclusions:**

These analyses suggest that the novel peer-referral strategy, the enhanced peer outreach approach, was effective in reaching female sex workeres in Côte d’Ivoire with demonstrated acquisition risks for HIV and who had not been effectively engaged by routine outreach approaches. Scaling up novel strategies such as enhanced peer outreach in the context of differentiated service models may be needed to optimize HIV prevention and treatment outcomes for key populations in Côte d’Ivoire.

## Introduction

Among countries in West and Central Africa, Côte d’Ivoire has one of the more broadly generalized HIV epidemics [1,2]. However, there is still a disproportionate burden of HIV among key populations, including female sex workers [3-6]. Compared to the HIV prevalence of 3.3% among women of reproductive age, the estimated prevalence for female sex workers is 7.5% [7]. Modeling studies [8,9] using data from Côte d’Ivoire have shown that allocating resources to improve HIV services to achieve the 90-90-90 targets set by the Joint United Nations Programme on HIV/AIDS (UNAIDS) among key populations by 2020 could avert 30% of new infections in the country [10]. Financing such HIV services was shown to be associated with an estimated 2% increase in the country’s current health care budget compared to an increase of approximately 14% for services which target Côte d’Ivoire’s entire population [9]. Addressing the HIV epidemic among key populations has been demonstrated to represent an essential and cost-effective component for the overall HIV response, particularly in the context of declining HIV funding in the country [9].

While HIV prevention and treatment services are available in existing health infrastructure, several factors interact to act as barriers to the uptake of these services. A systematic review [11] assessing the uptake of HIV testing among female sex workers found that, apart from demographic characteristics and risk behaviors, there were factors such as cost and time in accessing health care facilities, HIV testing policy shortcomings in confidentiality, and cost of health care impact uptake. Additionally, policies that specifically criminalize key populations and intersectional stigmas have resulted in limited coverage of these services among key populations [12,13]. Among female sex workers, legal barriers to practicing sex work have perpetuated physical and sexual violence. More than half of the female sex workers enrolled in a recent survey in Côte d’Ivoire reported experiencing physical violence and almost half had reported sexual violence [14]. Notably, violence against female sex workers impacts both the provision and uptake of HIV services, resulting in suboptimal health outcomes [11,14-16]. For female sex workers living with HIV, the intersectional stigmas associated with living with HIV and being a sex worker limit the uptake of and adherence to antiretroviral therapy, ultimately resulting in poor health outcomes among female sex workers and increasing the likelihood of onward transmission of HIV [17].

Given barriers to the uptake and provision of services for key populations in traditional health care settings, there have been widespread efforts to differentiate HIV prevention and treatment services for key populations, including the implementation of decentralized services. Differentiated HIV service delivery approaches aim to both be client-centered and to reduce the burden on the health system [18-21]. Elements of differentiated care include tailoring service frequency and intensity to the needs of clients, offering services in a range of locations, and task shifting services to different types of providers, including lay and peer providers who may themselves be members of key population communities [19,20]. While differentiated care models have mainly been used for decentralizing care for individuals stable on antiretroviral therapy or for antiretroviral therapy delivery, differentiated care may also be applied to different steps along the HIV care continuum [21-23].

In key population communities, differentiation efforts have included a focus on community-based and peer-driven approaches to meet and serve key population community members in environments that facilitate disclosure of key population-status and uptake of services by reducing stigma associated with accessing health care at facilities [21,24,25]. For example, in West Africa, HIV self-testing kits distributed through community outreach mechanisms have reached key populations who often had no history of HIV testing [26]. In addition, community-led peer education, condom distribution, and STI/HIV screening and treatment have all demonstrated impact in addressing traditionally marginalized communities at high risk of HIV [23,25]. Other studies have consistently shown that community-led programs that incorporate biomedical, behavioral, and structural approaches can support improved prevention and treatment outcomes [25].

Despite the promise that these decentralized community outreach approaches hold for addressing the epidemic among stigmatized populations, they may still not reach those who are most marginalized. Instead, they may repeatedly reach key populations who are already engaged while missing individuals who do not routinely present for prevention services during community-based outreach and, therefore, who may be at greater risk for HIV acquisition and transmission [24]. To address this limitation, researchers and program implementers are increasingly using approaches that leverage networks of key population members to engage with individuals who may not be easily accessible through traditional outreach methods. Methods which rely on social and sexual networks of key populations have been useful in recruiting individuals in research endeavors that aim to understand HIV-related behaviors among key populations. Respondent-driven sampling, a technique that uses a peer-to-peer referral mechanism to directly recruit individuals in a given social or sexual network, is an example of a peer-based recruitment method and is commonly used in research studies to recruit marginalized populations [27,28]. Where these approaches have been used as interventions by HIV programs, they have been shown to increase the uptake of HIV testing and have been effective in improving HIV and other STI case-finding [29,30].

The enhanced peer outreach approach is a programmatic strategy modeled after respondent-driven sampling developed by the United States Agency for International Development– and President's Emergency Plan For AIDS Relief–supported LINKAGES project to expand HIV services to key populations who have not previously engaged or who do not frequently engage with HIV programs [24,31]. A significant difference between enhanced peer outreach and research-oriented peer-based recruitment methods is that enhanced peer outreach aims to reach those at highest risk of HIV whereas other approaches aim to engage a more diverse sample of the key population community to increase the generalizability of the results. Similar to respondent-driven sampling though, enhanced peer outreach incorporates performance-based incentives and leverages social networks to improve HIV case-finding. Specifically, these approaches are based on the premise of shared social networks between individuals who have and have not been reached by routine HIV outreach. Leveraging these networks may result in increased HIV case-finding and linkage to care [24]. In this study, we describe characteristics of female sex workers reached by the LINKAGES project in Côte d’Ivoire through enhanced and routine peer outreach, and compare the performance of both strategies in improving case-finding and treatment initiation rates among female sex workers in Côte d’Ivoire between October 2017 and April 2018.

## Methods

### LINKAGES Project

LINKAGES is a global project that has worked in over 30 countries to reduce HIV transmission, increase case identification, improve treatment initiation and adherence, and achieve viral suppression among key populations [32]. The LINKAGES project works by partnering with governments, local community-based organizations, and the private sector to increase reach to key populations most at risk for HIV acquisition and transmission and expand access to comprehensive HIV prevention and treatment services [32].

The LINKAGES project routinely uses a community-led peer outreach approach to engage with key population members and provide a standardized HIV prevention package including testing, provision of condoms and lubricants, screening for other sexually transmitted infections, and counseling [31,32]. Peer outreach workers are themselves key population members and are recruited and trained by the local community-based organization implementing partners to deliver HIV prevention, testing, and adherence support services to other key population members who frequent hotspots or visit established clinical partners [24,31].

To expand service delivery to key populations who may not be reached through routine peer outreach, LINKAGES developed an enhanced peer outreach approach [24]. With enhanced peer outreach, trained peer outreach workers invite members of key populations to become peer mobilizers [24]. peer mobilizers, in turn, are given coupons by the peer outreach workers and asked to reach out to their social and sexual networks to encourage high-risk peers to get tested for HIV and seek other related services. Peer mobilizers and outreach workers are incentivized if the peers recruited are eligible for testing, accessed services, and agreed to an HIV test [24]. Although peer mobilizers are not formally trained, they are familiarized with the project, the enhanced peer outreach process, how to select potential participants, and how to distribute coupons [24]. Successfully recruited peers may themselves be encouraged to become peer mobilizers to bolster recruitment; however, it is not required. The coupons distributed have a unique code that links the tested peer to the outreach worker and peer mobilizer who gave out the coupon. The coupon code aids incentivization and helps track which networks were successful in recruiting newly diagnosed individuals. Eligibility for enhanced peer outreach is based on being a self-reported member of the key population, not having previously engaged with an HIV program, or not having been tested for HIV in the previous 3 months. Individuals are also considered to be eligible if they engaged with the program but had not been tested for HIV in the previous 3 months.

In Côte d’Ivoire, LINKAGES implemented the routine peer outreach approach year-round across 26 health districts while the enhanced peer outreach approach was conducted with 6 community-based organizations focused on female sex workers from March 19, 2018 to April 13, 2018 in 11 of the 26 health districts, including a mix of urban, peri-urban, and rural sites. Although community and facility testing are offered in the program in Côte d’Ivoire, testing was more frequently carried out in the community. Peer outreach workers and peer mobilizers received XOF 1500 (XOF: West African CFA Franc; approximately US $2.50) for every newly enrolled female sex worker who was eligible for HIV testing and, if the female sex worker was newly diagnosed with HIV, the peer outreach worker and the newly diagnosed female sex worker each received XOF 1500 ($2.50) to cover the cost of transportation to a treatment facility. The implementation details of enhanced peer outreach during the study period are presented in Table 1.

**Table 1:** Implementation details of the enhanced peer outreach approach in Côte d’Ivoire.

Characteristic		Details
Population		Female sex workers
Implementation dates		March 19, 2018 - April 13, 2018
**Sites**		
	n	11
	Health districts	Abidjan (2; Adjame and Attecoube), Anyama, Adzope, Daloa, Divo, Yamoussoukro, Bouake (3; Bouake Sud, Bouake Nord-Est and, Bouake Nord-Ouest)^a^, Bongouanou
	Type	Mix of urban, peri-urban, and rural
**Selection criteria**		
	Low case-finding rate	Sites recognized in previous quarters has having low case-finding rates and a large key population size were prioritized as they could be potential sites with a high unmet need for HIV testing services.
	Sites with higher case-finding rates	Sites with a higher number of HIV positive cases in the previous quarter were targeted to maximize reach, increase case-finding, and achieve saturation among the key population networks.
**Community-based organizations**		
	n	8
	Organizations	Blety, *Espace Confiance*, *Arc-En-Ciel Plus*, Alternative-CI, *Asapsu Yamoussoukro*, RSB, GBH, SAPharm
Peer outreach workers, n		78
Peer mobilizers, n		312
Why were these peer outreach workers and peer mobilizers chosen?		Based on performance (size of their network and ability to create more at risk key populations)
**Incentives**		
	For every new key population individual tested	XOF^b^ 1500
	Key population individual enrolled (tested HIV-positive)	XOF 3000 (1500 each to the individual and the peer outreach worker)

^a^South Bouake, Northeast Bouake, and Northwest Bouake, respectively.

^b^XOF: West African CFA franc.

### Data Collection

The data used in this study were deidentified routine programmatic data that had been collected as part of the LINKAGES project in Côte d’Ivoire. These data, representing a single time point for each client during the year, were extracted from routinely collected data entered into electronic databases from paper-based forms by implementing partners in 23 of the 26 health districts in Côte d’Ivoire between October 2017 and September 2018 (2018 fiscal year).

The standard forms used by LINKAGES’ implementing partners for program monitoring collected demographic information from clients reached, including age, gender, and key population member status. HIV risks of key populations were also characterized based on self-reported answers to questions related to sex work debut, number of sex acts in the past week, and condom use during their last sex encounter (Table 2). Data related to HIV testing, diagnosis, and treatment initiation were captured on the forms, as well as data to determine whether female sex workers had been reached through routine peer outreach or enhanced peer outreach.

**Table 2:** Risk evaluation categories for female sex workers reached by the LINKAGES program.

Criteria	Low risk	Medium risk	High risk
Age at sex work debut	25 years or older	Between 20 and 24 years	19 years or younger
Number of sex acts in previous week	Fewer than 7 sex acts	Between 7 and 21 sex acts	More than 21 sex acts
Condom use during last sex encounter	Yes	N/A	No

### Data Quality

At the end of each week, partners submitted data summaries to the LINKAGES central program office showing the number of key population individuals who were newly recruited, tested for HIV, newly diagnosed with HIV, and initiated on antiretroviral therapy. All data were validated on a regular basis following the established processes for data quality assurance. Weekly summary reports submitted by partners to the program office were compared with source documents such as registers and other intake forms in the partner office to ensure consistency. If discrepancies were observed in the data, then reasons for the discrepancies were ascertained and noted, and the data in the summary reports were adjusted to be consistent with those in the source documents.

### Statistical Analyses

All program participants included in these analyses were female sex workers who were reached in districts where both approaches were carried out. A female sex worker was defined as a cisgender woman whose majority of income in the past 12 months came from goods or money received in exchange for sex. In these analyses, individuals were tested if they reported no history of HIV tests, or if they had previous testing history but had not been tested for HIV in the previous 3 months, and they did not already know whether they were HIV positive. The HIV case-finding rate was defined as the proportion of previously undiagnosed HIV cases among those who were tested.

Demographic characteristics of all program clients included in our analyses, including age and geographic region, and HIV indicators of number newly tested, number of new diagnoses of HIV, prevalence, linkage to HIV treatment, and HIV treatment initiation were assessed using descriptive statistics. Pearson chi-square tests were used to compare the crude relationship between outreach approach and age, HIV testing history, HIV risk evaluation categories, HIV case-finding rate among those tested, linkage to care, and treatment initiation. A significance level of *P*<.05 was used for all analyses. Analyses were carried out using STATA (version 15; Stata Corp LLC) and Excel (2016, Microsoft Inc).

### Ethical Considerations

This analysis was reviewed by the FHI 360 Protection of Human Subjects Committee and classified as nonhuman research since the data did not contain individual identifiers.

## Results

### Overall Demographic Characteristics and HIV Outcomes

Data records were available for a total of 18,889 female sex workers who were reached by the LINKAGES program between October 2017 and April 2018. Data between May and September 2018 were excluded from these analyses because the time period coincided with a different outreach approach. Additionally, of the 23 health districts where the LINKAGES program was implemented, data for both enhanced peer outreach and routine outreach were available in 8: Adjame, Attecoube, Adzope, Divo, Bouake N-Ouest, Anyama, Daloa, and Yamoussoukro (Table 3). Therefore, the analyses were carried out using data for the 9761 women reached in these 8 health districts where the data from the 2 outreach approaches were available. Of the 9761 women, 83% (8049) were older than 20 years of age; the majority (8851/9761, 91%) of female sex workers in the analytical sample were tested in the 2018 fiscal year. Of the tested participants, 90% (7967/8851) tested negative while 8% (698/8851) were diagnosed with HIV. The prevalence of HIV in the sample, including previously diagnosed female sex workers, was 8.7% (847/9761). Among the female sex workers who were newly diagnosed with HIV, 81% (564/698) were linked to treatment while 76% (527/698) were initiated on treatment, representing 93% (527/564) of all those who were reported to be linked to care.

**Table 3:** Geographic distribution of a sample of female sex workers reached by enhanced peer outreach approach and routine peer-based outreach strategies in Côte d’Ivoire between October 2017 and April 2018.

Site	Total (N= 9761)	Enhanced peer outreach (n=2509), n (%)^a^	Routine peer outreach (n=7252), n (%)^a^
Adjame	2278	627 (27.5)	1651 (72.5)
Adzope	146	142 (97.3)	4 (2.7)
Anyama	319	24 (7.5)	295 (92.5)
Attecoube	1051	385 (36.6)	666 (63.4)
Bouake N-Ouest	996	458 (46.0)	538 (54.0)
Daloa	1651	299 (18.1)	1352 (81.9)
Divo	1783	392 (22.0)	1391 (78.0)
Yamoussoukro	1537	182 (11.8)	1355 (88.2)

^a^Percentage of women of the district total (in column 2).

### Comparison of Routine and Enhanced Peer Outreach Approaches

The age distribution and HIV outcomes of the female sex workers reached by enhanced peer outreach and the routine peer outreach in the districts where both approaches were carried out and included in the analysis are presented in Table 4. Standard outreach occurred throughout the duration of the study while enhanced peer outreach took place between March 19, 2018 and April 13, 2018. The total number of women reached in the districts where both approaches were carried out was 9761 and those reached by enhanced peer outreach represented 26% of the sample (2509/9761). Participants reached by enhanced peer outreach were more likely to be older than 20 (n=9761; χ^2^_1_=33.4; *P=*.001), and more likely to report that they had previously been tested for HIV (n=9761; χ^2^_1_=43.8; *P=*.001). Compared to female sex workers reached by standard peer outreach, women reached by enhanced peer outreach were more likely to be categorized as medium risk for numbers of sex acts in the past week, while those reached by the standard approach were more likely to be categorized as high risk for number of sex acts in the past week (n=9761; χ^2^_2_=306.6; *P=*.001). Women reached by enhanced peer outreach were also more likely to be categorized as medium or high risk for low age of sex work debut (n=9761; χ^2^_2_=341.1; *P=*.001) and high risk for condom use at last sex act (n=9761; χ^2^_1_=293.0; *P=*.001).

The HIV case-finding rate was higher with enhanced peer outreach compared to that of routine peer outreach (n=8665; χ^2^_1_=35.25; *P=*.001). Furthermore, compared to routine peer outreach, enhanced peer outreach was more likely to lead to linkage to treatment (n=698; χ^2^_1_=64.4; *P=*.001) and treatment initiation; however, the difference in treatment initiation was not statistically significant (n=698; χ^2^_1_=2.6; *P=*.11).

Additional sensitivity analyses during 2 different 26-day time periods (at the beginning of the fiscal year and 1 month before implementation of the enhanced peer outreach approach, excluding the month of March to prevent any potential overlap) were carried out (Multimedia Appendix 1). In the analysis using the beginning of the fiscal year (Multimedia Appendix 1), 3187 female sex workers were reached by both approaches with women reached by routine approach representing 21% (684/3187) of the sample. The demographic and behavioral risk distribution were similar to those observed in the main analyses (n=3078; χ^2^_1_=3.07; *P*<.001), and the case-finding rate for routine approach was 8.2% (47/567) while the rate for the enhanced peer outreach approach remained as 10.7% (268/2489). Linkage to treatment was high in both groups but remained higher for women reached by enhanced peer outreach (n=315; χ^2^_1_=8.64; *P*<.001). Treatment initiation was lower for women reached by enhanced peer outreach; however, the difference was not statistically significant (n=315; χ^2^_1_=8.64; *P*=.32). Similar results were observed using the 26-day time period before enhanced peer outreach approach implementation (Multimedia Appendix 1).

**Table 4:** Comparison of age and other HIV-related indicators by outreach approach.

Characteristics		Enhanced peer outreach (n=2509)	Routine peer outreach (n=7252)	Chi-square test (*df*)	*P* value
**Age categories**				33.4 (1)	.001
	≤20	345 (13.8)	1366 (18.8)		
	>20	2164 (86.3)	5885 (81.2)		
	Missing^a^	0	1 (<1)		
**Ever previously tested**				43.8 (1)	.001
	No	804 (32.0)	2855 (39.4)		
	Yes	1695 (67.6)	4352 (60.0)		
	Missing^a^	10 (0.4)	45 (0.6)		
**Number of sex acts in the past week**				306.6 (2)	.001
	Low risk (<7 sex acts )	529 (21.1)	2400 (33.1)		
	Medium risk (7-21 sex acts)	1367 (54.5)	2471 (34.1)		
	High risk (>21 sex acts)	581 (23.1)	2114 (29.2)		
	Missing^a^	32 (1.3)	267 (3.7)		
**Age of sex work debut**				341.1 (2)	.001
	Low risk (≥25 years old)	400 (15.9)	2524 (34.8)		
	Medium risk (20-24 years old)	1312 (52.3)	2781 (38.4)		
	High risk (≤19 years old)	762 (30.4)	1684 (23.2)		
	Missing^a^	35 (1.4)	263 (3.6)		
**Condom use at last sex act**				293.0 (1)	.001
	Low risk (yes)	1106 (44.1)	3883 (53.5)		
	High risk (no)	1387 (55.3)	3101 (42.8)		
	Missing^a^	16 (0.6)	268 (3.7)		
**HIV case-finding rate (n=8851)^b^**				35.3 (1)	.001
	Positive test result	269 (10.7)	429 (6.8)		
	Negative test result	2225 (89.2)	5742 (93.1)		
	Missing^a^	13 (0.52)	173 (2.7)		
**Linked to treatment (n=698)^c^**				64.4 (1)	.001
	No	11 (4.1)	123 (28.7)		
	Yes	258 (95.9)	306 (71.3)		
**Treatment initiation (n=698)^c^**				2.6 (1)	.11
	No	57 (21.2)	114 (26.6)		
	Yes	212 (78.8)	315 (73.4)		

^a^Missing categories excluded from statistical tests.

^b^Among female sex workers who were tested.

^c^Among female sex workers who were newly diagnosed with HIV.

## Discussion

### Principal Findings

In this study, 2 community-based outreach strategies were compared, both focused on engaging female sex workers in HIV testing and prevention services in Côte d’Ivoire. This study suggests that leveraging peer referral networks for HIV testing through the enhanced peer outreach approach may be an efficient strategy for reaching female sex workers for testing and diagnosis in Côte d’Ivoire. Results suggest that the enhanced peer outreach approach was able to reach female sex workers who had moderate to high HIV behavioral risks. There were improvements in case-finding rates for both approaches compared to those in the previous fiscal year (6% for enhanced peer outreach and 2% for standard outreach in the previous fiscal year) [24], and compared to the routine outreach, newly diagnosed individuals reached by enhanced peer outreach were more likely to be integrated into HIV care than those who were reached by the routine outreach approach.

The high case-finding rates observed in the program overall and in women reached by enhanced peer outreach compared to routine outreach, coupled with increases in case-finding from the previous year, highlight the potential utility of using targeted and differentiated service delivery approaches. Awareness of HIV status remains a critical step in meeting targets set by UNAIDS to reduce the global burden of HIV by 2030 [10]. Individuals who are diagnosed can be referred for antiretroviral therapy and those who are uninfected can benefit from appropriate risk-reduction counseling and prevention services such as the provision of condoms or pre-exposure prophylaxis treatments. Given that enhanced peer outreach was implemented to reach key populations who do not readily engage with health services, women diagnosed through the approach may have otherwise continued to transmit HIV or may have been identified at a later stage of disease progression, ultimately challenging HIV epidemic control. Mathematical modeling studies in Côte d’Ivoire suggest that HIV prevention and treatment services targeted to key populations could be the most cost-effective way to improve HIV control efforts in the country by averting up to 30% of new infections [9]. Since peer referral approaches have been well established as useful in recruiting marginalized populations for research studies and have previously been leveraged as intervention tools to influence HIV-related behavior and improve HIV outcomes [33,34], enhanced peer outreach could be used not only to increase testing uptake—an important application for differentiated care at the first step of the cascade—but also to promote HIV prevention knowledge, pre-exposure prophylaxis and condoms, thus modifying risk behaviors within networks [28,35]. However, these approaches have rarely been taken to scale limiting their public health impact to date [23,36]. Characterizing optimal implementation strategies to maximize impact and sustainability requires engagement with a broad range of stakeholders under the leadership of government and community actors [23,36].

While linkage to treatment and treatment initiation among female sex workers reached through LINKAGES was encouraging, 25% of the newly diagnosed women did not initiate treatment, indicating gaps in the second 95 target set by UNAIDS [10]. Given the high HIV prevalence observed in this sample, there is need for implementing novel linkage strategies together with the case-finding approaches evaluated here. An allowance of up to 1500 XOF for transportation to antiretroviral therapy facilities was provided to all female sex workers diagnosed with HIV who had been reached by enhanced peer outreach, and to the peer outreach worker involved in HIV diagnosis, to ensure linkage to treatment occurred; no difference was observed with treatment initiation by group, indicating that there might be a need for additional strategies to ensure those who link to treatment facilities initiate antiretroviral therapy and are retained. While the latest HIV treatment guidelines in Cote d’ Ivoire recommend immediate antiretroviral therapy for all persons diagnosed with HIV regardless of CD4 count, it is possible that budgetary constraints in clinical settings in Côte d’Ivoire hamper the implementation of immediate antiretroviral therapy initiation for some of those diagnosed [37].

All of the community-based organizations involved in the outreach approaches evaluated here were key population–led, or at least trained, to be competent in addressing the needs of key population reinforcing the importance of the role of key population–led organizations in improving HIV outcomes within their communities. With high levels of stigma still facing key populations in many regions of the world, key population–led organizations are increasingly recognized as central to reaching and effectively providing care to members of key population communities who may desire to access care but fear discrimination [38]. Moreover, evidence exists that community- and peer-driven approaches such as these are effective in improving not only testing but also in reducing incidence and improving treatment initiation and adherence [25,39].

These data were collected though programmatic surveillance and represent real-world settings. This study is an example of how data collected by community-based HIV programs may be used to answer epidemiological questions. Dedicated epidemiological studies can be prohibitively expensive and leveraging programmatic data collection to understand populations and inform program implementation provides an opportunity for efficiently and effectively addressing the epidemic. Moreover, as funding to carry out research studies becomes limited and the HIV epidemic continues, there is a need to better utilize routinely collected program data to address existing knowledge gaps [40].

Our findings should be interpreted with consideration of several limitations. The data used in this evaluation were program data. While program data may fill existing knowledge gaps, these data were not collected for research purposes. Therefore, lapses in information and inconsistencies in data collection or data entry may have affected our results. Even with routine data quality assurance measures taken by the program, discrepancies in data entry and missing data were common, limiting our ability to use some variables that may have contributed to the rigor of these analyses and impacting the interpretation of our findings. Furthermore, these data were collected in one time period in one fiscal year. It is possible that similar results may not be observed in other time periods. Comparisons of findings at other time points could have strengthened these analyses. However, results from a recent study [24] using data from the previous fiscal year indicate an increasing trend in new HIV diagnoses. Given that the enhanced peer outreach approach was carried during a 1-month period of the fiscal year, an analysis comparing both approaches within the same time period would have been preferable. However, focusing on the part of the program to reach more high-risk individuals during the time period that enhanced peer outreach was carried out meant that virtually all key populations reached during the time period were recruited through the enhanced peer outreach approach limiting our ability to effectively carry out a comparison during the same time period. Nevertheless, we have included sensitivity analyses using the 26-day period at the beginning of the fiscal year and an additional 26-day period before the implementation of enhanced peer outreach approach as comparisons, and the interpretations of our findings remained the same. Incentives provided to peer outreach workers and peer mobilizers could have introduced participation bias and differential recruitment by approach impacting the representativeness of our findings and enhanced peer outreach approach if implemented as an intervention in Côte d’Ivoire. Thus, implementation could be carried out systematically and targeted in only populations where effectiveness has been shown. Additionally, monetary incentives could be substituted with small nonmonetary prizes which are potentially more scalable. Engagement with local stakeholders is needed to ensure the most cost-effective approach to scaling. The LINKAGES program in Côte d’Ivoire is a community-based program and only followed key population peers until linkage to treatment. Other partners tracked and monitored viral load in persons living with HIV after antiretroviral therapy initiation; therefore, the ability of the LINKAGES to monitor treatment initiation is limited. The lack of statistical significance in treatment initiation comparing enhanced peer outreach approach and standard outreach (n=698; χ^2^_1_=2.6; *P=*.11) may therefore be due to gaps in data. Findings may not be generalizable to other regions, and since the program participants represented in our data were female sex workers, our results may not apply to other key populations. However, peer referral has been shown to be effective for reaching other key populations including men who have sex with men and people who inject drugs [33,41,42]. The data used here to evaluate the risk profiles of the female sex workers were limited due to high levels of missing data. Furthermore, responses to the individual questions which made up the risk evaluations for women reached by the routine peer approach were only available as categorized variables therefore misclassification of risk could have occurred. Finally, all behavioral data collected were self-reported by clients of the program, making the findings susceptible to recall and social desirability biases.

### Conclusion

In the context of a large-scale HIV response, there has only been a 32% decrease in new infections over the last nine years in Côte d’Ivoire. Epidemiologic and cost-effectiveness data suggest that targeted approaches may support increased impact within existing budget envelopes. These analyses demonstrated that the peer referral–based strategy—enhanced peer outreach—was effective in reaching those who are at higher risk for HIV acquisition and transmission and who may not frequently utilize existing HIV services. Achieving greater gains with increasingly limited resources necessitates more widespread implementation of novel approaches that engage communities traditionally disconnected from HIV prevention and treatment services in Côte d’Ivoire.

## Appendix

Multimedia Appendix 1 Sensitivity analyses using two different time points for comparison.

## Abbreviations

XOF: West African CFA Franc
HIV: Human immunodeficiency virus
STI: sexually transmitted infection
UNAIDS: Joint United Nations Programme on HIV/AIDS

## References

[1] Maheu-Giroux M, Baral S, Vesga JF, Diouf D, Diabaté S, Alary M, Abo K, Boily M (2018). Anal intercourse among women working in the sex industry in Côte d'Ivoire: prevalence, determinants, and model-based estimates of the population-level impact on HIV transmission. Am J Epidemiol.

[2] Maheu-Giroux M, Vesga JF, Diabaté S, Alary M, Baral S, Diouf D, Abo K, Boily M (2017). Changing dynamics of HIV transmission in Côte d'Ivoire: modeling who acquired and transmitted infections and estimating the impact of past HIV interventions (1976-2015). J Acquir Immune Defic Syndr.

[3] Beyrer Chris, Baral Stefan D, van Griensven Frits, Goodreau Steven M, Chariyalertsak Suwat, Wirtz Andrea L, Brookmeyer Ron (2012). Global epidemiology of HIV infection in men who have sex with men. Lancet.

[4] Baral S, Sifakis F, Cleghorn F, Beyrer C (2007). Elevated risk for HIV infection among men who have sex with men in low- and middle-income countries 2000-2006: a systematic review. PLoS Med.

[5] Baral S, Beyrer C, Muessig K, Poteat T, Wirtz A, Decker Michele R, Sherman Susan G, Kerrigan Deanna (2012). Burden of HIV among female sex workers in low-income and middle-income countries: a systematic review and meta-analysis. Lancet Infect Dis.

[6] The gap report. UNAIDS.

[7] Côte d'Ivoire. UNAIDS.

[8] Maheu-Giroux M, Vesga J, Diabaté Souleymane, Alary M, Baral S, Diouf Daouda, Abo Kouamé, Boily Marie-Claude (2017). Population-level impact of an accelerated HIV response plan to reach the UNAIDS 90-90-90 target in Côte d'Ivoire: insights from mathematical modeling. PLoS Med.

[9] Maheu-Giroux M, Diabaté Souleymane, Boily M, Jean-Paul N, Vesga JF, Baral S, Abo K, Wognin V, Diouf D, Alary M (2019). Cost-effectiveness of accelerated HIV response scenarios in Côte d'Ivoire. J Acquir Immune Defic Syndr.

[10] 90-90-90: an ambitious treatment target to help end the AIDS epidemic. UNAIDS.

[11] Tokar A, Broerse JEW, Blanchard J, Roura M (2018). HIV testing and counseling among female sex workers: a systematic literature review. AIDS Behav.

[12] Schwartz SR, Nowak RG, Orazulike I, Keshinro B, Ake J, Kennedy S, Njoku O, Blattner WA, Charurat ME, Baral SD, TRUST Study Group (2015). The immediate eff ect of the Same-Sex Marriage Prohibition Act on stigma, discrimination, and engagement on HIV prevention and treatment services in men who have sex with men in Nigeria: analysis of prospective data from the TRUST cohort. Lancet HIV.

[13] Crowell TA, Keshinro B, Baral SD, Schwartz SR, Stahlman S, Nowak RG, Adebajo S, Blattner WA, Charurat ME, Ake JA (2017). Stigma, access to healthcare, and HIV risks among men who sell sex to men in Nigeria. J Int AIDS Soc.

[14] Lyons CE, Grosso A, Drame FM, Ketende S, Diouf D, Ba I, Shannon K, Ezouatchi R, Bamba A, Kouame A, Baral S (2017). Physical and sexual violence affecting female sex workers in Abidjan, Côte d'Ivoire: prevalence, and the relationship with the work environment, HIV, and access to health services. J Acquir Immune Defic Syndr.

[15] Lasater ME, Grosso A, Ketende S, Lyons C, Pitche VP, Tchalla J, Anato S, Sodji D, Nadedjo F, Baral S (2019). Characterising the relationship between migration and stigma affecting healthcare engagement among female sex workers in Lomé, Togo. Glob Public Health.

[16] Wanyenze RK, Musinguzi G, Kiguli J, Nuwaha F, Mujisha G, Musinguzi J, Arinaitwe J, Matovu JKB (2017). "When they know that you are a sex worker, you will be the last person to be treated": Perceptions and experiences of female sex workers in accessing HIV services in Uganda. BMC Int Health Hum Rights.

[17] Mtetwa S, Busza J, Chidiya S, Mungofa S, Cowan F (2013). "You are wasting our drugs": health service barriers to HIV treatment for sex workers in Zimbabwe. BMC Public Health.

[18] Consolidated guidelines on HIV prevention, diagnosis, treatment and care for key populations. World Health Organization.

[19] Macdonald Virginia, Verster Annette, Baggaley Rachel (2017). A call for differentiated approaches to delivering HIV services to key populations. J Int AIDS Soc.

[20] Baggaley R, Armstrong A, Dodd Z, Ngoksin E, Krug A (2015). Young key populations and HIV: a special emphasis and consideration in the new WHO Consolidated Guidelines on HIV Prevention, Diagnosis, Treatment and Care for Key Populations. J Int AIDS Soc.

[21] Grimsrud A, Bygrave H, Doherty M, Ehrenkranz P, Ellman T, Ferris Robert, Ford Nathan, Killingo Bactrin, Mabote Lynette, Mansell Tara, Reinisch Annette, Zulu Isaac, Bekker Linda-Gail (2016). Reimagining HIV service delivery: the role of differentiated care from prevention to suppression. J Int AIDS Soc.

[22] Schwartz Sheree R, Baral Stefan (2019). Remembering individual perspectives and needs in differentiated HIV care strategies. BMJ Qual Saf.

[23] Ehrenkranz P, Grimsrud A, Rabkin M (2019). Differentiated service delivery: navigating the path to scale. Curr Opin HIV AIDS.

[24] Lillie T, Persaud N, DiCarlo M, Gashobotse D, Kamali D, Cheron Magda, Nishimoto Lirica, Akolo Christopher, Mahler Hally R, Au Maria C, Wolf R Cameron (2019). Reaching the unreached: performance of an enhanced peer outreach approach to identify new HIV cases among female sex workers and men who have sex with men in HIV programs in West and Central Africa. PLoS One.

[25] Wirtz AL, Pretorius C, Beyrer C, Baral S, Decker MR, Sherman SG, Sweat M, Poteat T, Butler J, Oelrichs R, Semini I, Kerrigan D (2014). Epidemic impacts of a community empowerment intervention for HIV prevention among female sex workers in generalized and concentrated epidemics. PLoS One.

[26] Lyons CE, Coly K, Bowring AL, Liestman B, Diouf D, Wong VJ, Turpin G, Castor D, Dieng P, Olawore O, Geibel S, Ketende S, Ndour C, Thiam S, Touré-Kane Coumba, Baral SD (2019). Use and acceptability of HIV self-testing among first-time testers at risk for HIV in Senegal. AIDS Behav.

[27] Heckathorn D (1997). Respondent-driven sampling: a new approach to the study of hidden populations. Social Problems.

[28] Stahlman S, Johnston LG, Yah C, Ketende S, Maziya S, Trapence G, Jumbe V, Sithole B, Mothopeng T, Mnisi Z, Baral S (2016). Respondent-driven sampling as a recruitment method for men who have sex with men in southern sub-Saharan Africa: a cross-sectional analysis by wave. Sex Transm Infect.

[29] Pines HA, Goodman-Meza D, Pitpitan EV, Torres K, Semple SJ, Patterson TL (2016). HIV testing among men who have sex with men in Tijuana, Mexico: a cross-sectional study. BMJ Open.

[30] Rosenberg NE, Kamanga G, Pettifor AE, Bonongwe N, Mapanje C, Rutstein SE, Ward M, Hoffman IF, Martinson F, Miller WC (2014). STI patients are effective recruiters of undiagnosed cases of HIV: results of a social contact recruitment study in Malawi. J Acquir Immune Defic Syndr.

[31] LINKAGES enhanced peer outreach approach (EPOA): implementation guide. FHI 360.

[32] LINKAGES project. USAID.

[33] Baral SD, Ketende S, Schwartz S, Orazulike I, Ugoh K, Peel SA, Ake J, Blattner W, Charurat M (2015). Evaluating respondent-driven sampling as an implementation tool for universal coverage of antiretroviral studies among men who have sex with men living with HIV. J Acquir Immune Defic Syndr.

[34] Mavhu W, Willis N, Mufuka J, Bernays S, Tshuma M, Mangenah C, Maheswaran H, Mangezi W, Apollo T, Araya R, Weiss HA, Cowan FM (2020). Effect of a differentiated service delivery model on virological failure in adolescents with HIV in Zimbabwe (Zvandiri): a cluster-randomised controlled trial. Lancet Glob Health.

[35] Amirkhanian YA (2014). Social networks, sexual networks and HIV risk in men who have sex with men. Curr HIV/AIDS Rep.

[36] Ehrenkranz PD, Calleja JM, El-Sadr W, Fakoya AO, Ford N, Grimsrud A, Harris KL, Jed SL, Low-Beer D, Patel SV, Rabkin M, Reidy WJ, Reinisch A, Siberry GK, Tally LA, Zulu I, Zaidi I (2018). A pragmatic approach to monitor and evaluate implementation and impact of differentiated ART delivery for global and national stakeholders. J Int AIDS Soc.

[37] Ouattara EN, MacLean RL, Danel C, Borre ED, Gabillard D, Huang M, Moh R, Paltiel AD, Eholié SP, Walensky RP, Anglaret X, Freedberg KA (2019). Cost-effectiveness and budget impact of immediate antiretroviral therapy initiation for treatment of HIV infection in Côte d'Ivoire: A model-based analysis. PLoS One.

[38] Beyrer C, Baral S, Kerrigan D, El-Bassel N, Bekker L, Celentano DD (2011). Expanding the space: inclusion of most-at-risk populations in HIV prevention, treatment, and care services. J Acquir Immune Defic Syndr.

[39] Mulongo S, Kapila G, Hatton T, Canagasabey D, Arney J, Kazadi T, Scott LM, Colfax G (2015). Applying innovative approaches for reaching men who have sex with men and female sex workers in the Democratic Republic of Congo. J Acquir Immune Defic Syndr.

[40] McGillen JB, Sharp A, Honermann B, Millett G, Collins C, Hallett TB (2017). Consequences of a changing US strategy in the global HIV investment landscape. AIDS.

[41] Uusküla A, Johnston L, Raag M, Trummal A, Talu A, Des Jarlais Don C (2010). Evaluating recruitment among female sex workers and injecting drug users at risk for HIV using respondent-driven sampling in Estonia. J Urban Health.

[42] Kan M, Garfinkel DB, Samoylova O, Gray RP, Little KM (2018). Social network methods for HIV case-finding among people who inject drugs in Tajikistan. J Int AIDS Soc.

