# Supplementary material for: Peer Recruitment Strategies for Female Sex Workers Not Engaged in HIV Prevention and Treatment Services in Côte d’Ivoire: Program Data Analysis
Source: JMIR Public Health Surveill. 2020 Oct 1;6(4):e18000. doi: 10.2196/18000 (PMC7563635; doi:10.2196/18000)
Supplement: Multimedia Appendix 1 [file publichealth_v6i4e18000_app1.docx]

Supplemental Table 1: Comparison using the same twenty-six-day length of time. Time period for routine peer approach was at the beginning of the fiscal year, between (October 2, 2017 and October 27, 2017).

| **Characteristics (N=3,187)** | **EPOA (N=2,503)** | **Routine Peer Approach (N=684)** | **Chi-square Tests(df)** |
| --- | --- | --- | --- |
| **Age categories**  20 or younger  Older than 20 | 345 (13.8)  2,158 (86.2) | 163 (28.8)  521 (76.2) | χ2 (1) = 40.47  p **<.001** |
| **Ever previously tested**  No  Yes  Missing | 804 (32.0)  1,689 (67.5)  10 (0.4) | 241(35.2)  423 (61.8)  20 (2.9) | χ2 (1 )= 3.87  p **<.001** |
| **Number of sex acts in the past week**  Low risk (< 7 sex acts )  Medium risk (7-21 sex acts)  High risk (> 21 sex acts)  Missing | 527 (21.1)  1,365 (54.5)  579 (23.1)  32 (1.3) | 90 (13.2)  242 (35.4)  277 (32.4)  267 (3.7) | χ2 (2) = 118.46  p **< .001** |
| **Age of sex work debut**  Low risk ( ≥ 25 years old)  Medium risk (20-24 years old)  High risk (≤ 19 years old)  Missing | 397 (15.9)  1,310 (52.3)  761 (30.4)  35 (1.4) | 262 (38.3)  192 (28.1)  155 (22.7)  75 (11.0) | χ2 (2) = 216.78  p **< .001** |
| **Condom use at last sex act**  Low risk (Yes)  High Risk (No)  Missing | 1,103 (44.1)  1,384 (55.3)  16 (0.6) | 108 (15.8)  504 (73.7)  72 (10.5) | χ2 (1) = 147.11  p **< .001** |
| **HIV Case finding rate (N=3,078)^b^**  Positive test result  Negative test result  Missing | 268 (10.7)  2,221 (89.2)  12 (0.5) | 47 (8.2)  520 (91.7)  10 (1.7) | χ2 (1) = 3.07  p **< .001** |
| **Linked to treatment ((N=315)^c^**  No  Yes | 11 (4.1)  257 (95.9) | 7 (14.9)  40 (85.1) | χ2 (1) = 8.64  p **= .003** |
| **Treatment initiation (N= 315)^c^**  No  Yes | 57 (21.3)  211 (78.7) | 7 (14.9)  40 (85.1) | χ2 (1) = 8.64  p = .316 |

^a^ Missing category excluded from statistical tests

^b^ Among FSW who were tested

^c^ Among FSW who were newly diagnosed with HIV

Bolded represents p <0.05

Supplemental table 2: Comparison using the same twenty-six-day length of time. Time period for routine peer approach was a twenty-six-day period before the implementation of EPOA, between (February 01, 2018 and February 26, 2018).

| **Characteristics (N=4,182)** | **EPOA (N=2,503)** | **Routine Peer Approach (N=1,679)** | **Chi-square Tests (df)** |
| --- | --- | --- | --- |
| **Age categories**  20 or younger  Older than 20 | 345 (13.8)  2,158 (86.2) | 315 (18.8)  1,364 (81.2) | χ2 (1) = 18.74  p **< .001** |
| **Ever previously tested**  No  Yes  Missing | 804 (32.1)  1,689 (67.5)  10 (0.4) | 771 (45.9)  888 (52.9)  20 (1.2) | χ2 (1) = 85.59  p **< .001** |
| **Number of sex acts in the past week**  Low risk (< 7 sex acts )  Medium risk (7-21 sex acts)  High risk (> 21 sex acts)  Missing | 527 (21.1)  1,365 (54.5)  579 (23.1)  32 (1.3) | 510 (30.4)  650 (38.7)  471 (28.1)  48 (2.9) | χ2 (2) = 97.16  p **< .001** |
| **Age of sex work debut**  Low risk ( ≥ 25 years old)  Medium risk (20-24 years old)  High risk (≤ 19 years old)  Missing | 397 (15.9)  1,310 (52.3)  761 (30.4)  35 (1.4) | 433 (25.8)  714 (42.5)  484 (28.8)  48 (2.9) | χ2 (2) = 70.73  p **< .001** |
| **Condom use at last sex act**  Low risk (Yes)  High Risk (No)  Missing | 1,103 (44.1)  1,384 (55.3)  16 (0.6) | 753 (44.9)  881 (52.5)  45 (2.7) | χ2 (1) = 1.20  p = .274 |
| **HIV Case finding rate (N=3,999)^b^**  Positive test result  Negative test result  Missing | 268 (10.7)  2,221 (88.8)  12 (0.5) | 140 (9.4)  1,346 (89.9)  12 (0.8) | χ2 (1) = 1.83  p = .176 |
| **Linked to treatment ((N=408)^c^**  No  Yes | 11 (4.1)  257 (95.9) | 20 (14.3)  10 (85.7) | χ2 (1) = 13.58  p **< .001** |
| **Treatment initiation (N= 408)^c^**  No  Yes | 57 (21.3)  211 (78.7) | 20 (14.3)  120 (85.7) | χ2 (1) = 2.93  p = .087 |

^a^ Missing category excluded from statistical tests

^b^ Among FSW who were tested

^c^ Among FSW who were newly diagnosed with HIV

Bolded represents p <0.05
